# Respiratory viruses dynamics and interactions: ten years of surveillance in central Europe

**DOI:** 10.1186/s12889-022-13555-5

**Published:** 2022-06-11

**Authors:** Gibran Horemheb-Rubio, Ralf Eggeling, Norbert Schmeiβer, Nico Pfeifer, Thomas Lengauer, Barbara C. Gärtner, Christiane Prifert, Matthias Kochanek, Christoph Scheid, Ortwin Adams, Rolf Kaiser, Respiratory Virus Network, Rolf Kaiser, Barbara C. Gärtner, Benedikt Weissbrich, Ortwin Adams, Annemarie Berger, Katrin Palupsky, Daniela Huzly, Carsten Tiemann, Wegene Borena, Andreas Lindauer, Uwe Gerd Liebert, Hans-Joachim Siemens, Jörg Hofmann, Anna-Maria Eis-Hübinger, Hajo Grundmann, Astrid Kehlen, Albrecht Oehme, Paul Schnitzler, Joachim Kühn, Albert Heim, Andreas Sauerbrei, Barbara Schmidt, Robert Beck, Dieter Hoffmann, Detlef Michel, Hans Nitschko, Christian Aepinus, Jens Dreier, Elisabeth Puchhammer-Stoeckl, Theresa Popow Kaup, Monika Redlberger, Harald Kessler, Martin Obermeier, Kerstin Weise, Patricia Bartsch, Annette Devide, Bert Niesters, Michael Kleines, Andi Krumbholz, Thomas Meyer, Peter Gohl, Christian G. Schüttler, Meri Gorgievski, Andres Anton Pagarolas, Wolfgang Gulich, Thomas Ziegler, Babett Wintsche, Marcena Griego, Walter Bossart

**Affiliations:** 1 Institute of Virology, Faculty of Medicine and University Hospital Cologne, Köln, Germany; 2 Department of Infectious Diseases, Instituto Nacional de Ciencias Médicas Y Nutrición Salvador Zubirán, Mexico City, Mexico; 3 DZIF, Center for Infection Research, partner site Cologne Bonn, Cologne, Germany; 4 Methods in Medical Informatics, Department of Computer Science, University of Tübingen, Tübingen, Germany; 5 Medeora GmbH, Cologne, Germany; 6 Faculty of Medicine, University of Tübingen, Tübingen, Germany; 7 German Center for Infection Research, Partner Site Tübingen, Tübingen, Germany; 8 Computational Biology, Max Planck Institute for Informatics, Saarland Informatics Campus, Saarbrücken, Germany; 9 Institute of Medicine Microbiology and Hygiene, University of the Saarland Kirrberger Homburg/Saar, Homburg, Germany; 10 Faculty of Medicine, Institute for Virology and Immunobiology, Würzburg University, Würzburg, Germany; 11 University of Cologne, Department I of Internal Medicine, Center for Integrated Oncology, Aachen Bonn Cologne Düsseldorf, Cologne, Germany; 12 University of Düsseldorf, Medical Faculty, Institute for Virology, Düsseldorf, Germany

**Keywords:** Respiratory viruses, Coinfection, Seasonality, Surveillance, Viral exclusion

## Abstract

**Background:**

Lower respiratory tract infections are among the main causes of death. Although there are many respiratory viruses, diagnostic efforts are focused mainly on influenza. The Respiratory Viruses Network (RespVir) collects infection data, primarily from German university hospitals, for a high diversity of infections by respiratory pathogens. In this study, we computationally analysed a subset of the RespVir database, covering 217,150 samples tested for 17 different viral pathogens in the time span from 2010 to 2019.

**Methods:**

We calculated the prevalence of 17 respiratory viruses, analysed their seasonality patterns using information-theoretic measures and agglomerative clustering, and analysed their propensity for dual infection using a new metric dubbed average coinfection exclusion score (ACES).

**Results:**

After initial data pre-processing, we retained 206,814 samples, corresponding to 1,408,657 performed tests. We found that Influenza viruses were reported for almost the half of all infections and that they exhibited the highest degree of seasonality. Coinfections of viruses are frequent; the most prevalent coinfection was rhinovirus/bocavirus and most of the virus pairs had a positive ACES indicating a tendency to exclude each other regarding infection.

**Conclusions:**

The analysis of respiratory viruses dynamics in monoinfection and coinfection contributes to the prevention, diagnostic, treatment, and development of new therapeutics. Data obtained from multiplex testing is fundamental for this analysis and should be prioritized over single pathogen testing.

**Supplementary Information:**

The online version contains supplementary material available at 10.1186/s12889-022-13555-5.

## Background

The current COVID-19 pandemic prominently demonstrates the serious threat posed by respiratory infections, not only for the health of individuals, but also for the stability of modern society, in general. While SARS-CoV-2 infections are currently extensively recorded and analysed, future studies must encompass the full breadth of respiratory viruses as has been done in the past. Even before the pandemic, lower track respiratory infections were among the main causes of death in children and adults [1, 2]. Influenza infection killed between 250,000 and 500,000 people annually, 152,000 deaths were reported in Europe in the 2017–2018 season [3]. In Germany, during the 2018–2019 season, 182,000 influenza-positive tests were confirmed, including 40,000 from inpatients [4].

In 2009 the Respiratory Viruses Network (RespVir www.clinical-virology.net) was founded as an initiative of a Clinical Virology group within the German Virology Society (GfV). The purpose of RespVir is to record respiratory infections in an online database [5], providing clinicians with up-to-date information about circulating pathogens. The RespVir database contains mainly registries from inpatients data reported by 47 laboratories from university hospitals and a few private. These institutions are located primarily in Germany, Austria, and Switzerland, collecting data from central Europe. Over 12 years, RespVir has analysed more than 280,000 samples with respect to 25 respiratory pathogens (17 viruses and 8 bacteria). Among these years RespVir had obtained data on causal agents of respiratory infections.

RespVir includes records of samples from all patients with respiratory symptoms, sent in by clinicians requesting a diagnosis. Independent of the diagnostic hypothesis of the clinician, each sample was tested in a multiplex manner covering a maximum of 17 respiratory viruses, depending on test availability of each laboratory.

In this study, our aims were (i) to describe the prevalence and seasonal variation (seasonality) for each pathogen, (ii) to assess the prevalence of coinfections and (iii) to determine the rate of exclusion or affinity for pairwise coinfections.

After filtering registries with incomplete data and a post-hoc data quality control. To accomplish our objectives, we performed the analysis in a subset of the RespVir database including 17 different viral pathogens covering the time span from 2010 to 2019.

We observed that 48.64% of all reported respiratory infections are caused by influenza virus. We found four general seasonality patterns. Each of the 17 viruses belongs to one of these patterns. Stratification across years shows biennial seasonality patterns for some viruses, indicating infection peaks every other season. We further observed that coinfections do not occur statistically independently, but that for most virus pairs coinfection is far less frequent than expected by chance.

## Methods

### Samples and data collection

**Fig. 1:**
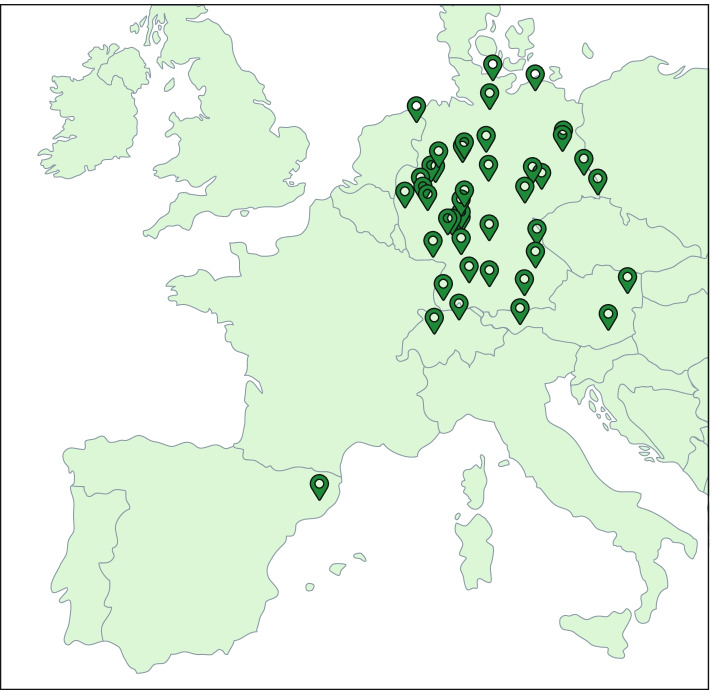
Distribution of RespVir Network. The figure shows a European map with the location of the 47 laboratory members of RespVir Network. These laboratories are located in the following countries: Germany, Austria, Switzerland, Netherlands, and Spain

Since November 2009, the RespVir network (Fig. 1) has collected multiplex test records for 17 virus infections from patients that showed respiratory infection symptoms. The records stem from various 47 sites, according to the sites’ test availability. The tests used by some sites do not differentiate between certain virus types or subtypes: (i) FLUA-generic, which cannot distinguish between influenza A H1N1 and H3N2, (ii) HPIV-generic, which cannot differentiate between parainfluenzas 1, 2, 3 or 4, (iii) HCoV-generic which cannot differentiate between the human coronaviruses HCoV-OC43, HCoV-NL63, HCoV-229E, and HCoV-HKU1, and (iv) RV/EV which does not differentiate between rhinovirus and enterovirus (Table 1). Each RespVir member site submitted a file that includes the date of sampling, the tests performed and their results to a data base manager, who fed the data to the database.

**Table 1:** Prevalence of Circulating Viruses

Prevalence of Circulating Viruses								
Tests Used for Viral Infection Diagnostic		Tested		Negatives		Positives		
tested virus	Abbreviation	Number	Proportion	Number	Percentage	Number	Percentage	Proportion
Influenza A (H3N2)	FLUA(H3N2)	93,848	6.66%	81,711	87.07%	12,137	12.93%	7.93%
Influenza A (H1N1)	FLUA(H1N1)	65,468	4.65%	60,602	92.57%	4,866	7.43%	3.18%
Non-differentiated Influenza A (H1N1 and H3N2)	FLUA-generic	96,711	6.87%	64,907	67.11%	31,804	32.89%	20.79%
Influenza B	FLUB	168,628	11.97%	143,013	84.81%	25,615	15.19%	16.74%
Parainfluenza 1	HPIV-1	77,011	5.47%	75,848	98.49%	1,163	1.51%	0.76%
Parainfluenza 2	HPIV-2	76,406	5.42%	75,431	98.72%	975	1.28%	0.64%
Parainfluenza 3	HPIV-3	77,981	5.54%	73,380	94.10%	4,601	5.90%	3.01%
Parainfluenza 4	HPIV-4	45,825	3.25%	44,978	98.15%	847	1.85%	0.55%
Non-differentiated Parainfluenza (1,2,3, and 4)	HPIV-generic	21,949	1.56%	20,882	95.14%	1,067	4.86%	0.70%
Metapneumovirus	HMPV	86,107	6.11%	80,858	93.90%	5,249	6.10%	3.43%
Respiratory Syncytial Virus	HRSV	97,976	6.96%	78,015	79.63%	19,961	20.37%	13.05%
Rhinovirus	RV	74,061	5.26%	53,150	71.77%	20,911	28.23%	13.67%
Enterovirus	EV	63,444	4.50%	59,377	93.59%	4,067	6.41%	2.66%
Non-differentiated Picornaviruses (Rhinovirus and Enterovirus)	RV/EV	8,604	0.61%	6,823	79.30%	1,781	20.70%	1.16%
Adenovirus	HAdV	80,593	5.72%	73,611	91.34%	6,982	8.66%	4.56%
Coronavirus OC43	HCoV-OC43	63,523	4.51%	60,818	95.74%	2,705	4.26%	1.77%
Coronavirus E229	HCoV-E229	59,369	4.21%	58,132	97.92%	1,237	2.08%	0.81%
Coronavirus NL63	HCoV-NL63	61,455	4.36%	59,922	97.51%	1,533	2.49%	1.00%
Coronavirus HKU1	HCoV-HKU1	17,013	1.21%	16,812	98.82%	201	1.18%	0.13%
Non-differentiated Coronaviruses (OC43, E229, NL63 and HKU1)	HCoV-generic	7,628	0.54%	6,909	90.57%	719	9.43%	0.47%
Bocavirus	HBoV	65,057	4.62%	60,474	92.96%	4,583	7.04%	3.00%
**Total**		**1,408,657**	**100%**	**1,255,653**		**153,004**		**100%**

Table 1 Prevalence of Circulating Viruses. The table describes the 21 tests to detect 17 respiratory viruses, indicating the name of the test performed, the abbreviation for this study. The table also shows the number of tests performed, tests with negatives outcome and tests with positive outcome of each specific test type. The proportion indicates the percentage of all tests in each category (tested and positives). The percentage indicates the negativity and positivity percentage of each test type

### Database pre-processing

The database required a pre-processing of the data. This process consisted in 1) filtering all incomplete data records before further analysis, and 2) a post-hoc curation.

After manual inspection of the data records, we found that most of the sites reported coinfection rates below 0.01 of any virus pairs. There were few sites (up to 4 out of 47, depending on the analysed virus pairs) reporting coinfection rates above 0.01. Due to the possibility of typographic errors and to avoid overestimation of the coinfection rates, we analysed only coinfections for each virus pairs reported below 0.01 threshold per site. Of note, this threshold also did not significantly affect the results.

After the pre-processing we split the database in two for the analysis. The first part of the analysis was performed on data records from monoinfections. Therefore, we filtered the data records for monoinfection. The second analysis was coinfections. For the coinfection analysis we took the records from samples tested for multiple viruses and exclusively evaluated coinfections with two viruses.

### Seasonality profiles

We computed the *seasonality profile* of a virus by stratifying the number of positive tests from 2010 to 2019 by month and normalizing these month-specific infection counts. To quantify the *degree of the seasonality* of each virus, we computed the Kullback–Leibler divergence (KLD) of the seasonality profile to a uniform distribution [6]. Hence, a value of zero corresponds to uniform prevalence over the year, i.e., no seasonal variation. To compare the seasonality profiles of the 17 pathogens among each other, we computed the Jensen-Shannon divergence (JSD) [7] and applied agglomerative clustering with average linkage.

We also computed *year-specific seasonality profiles* for each year from 2010 to 2019. We clustered these profiles according to their JSD to compare the seasonality profiles over different years. In addition, we repeated the year-specific analysis using the positivity percentage instead of the absolute counts of positive tests.

### Coinfections prevalence and virus pair relations

For studying coinfections, we considered the 17 viruses corresponding to 21 tests including the non-differentiating tests (FLUA-generic, HPIV-generic, RV/EV and HCoV-generic). While theoretically 210 combinations of the 21 tests are possible, we excluded combinations of non-differentiating tests with their more specific counterparts, such as FLUA(H3N2) combined with FLUA-generic. Finally, 197 valid combinations remained.

For assessing the tendency of each virus pair to exclude each other or coinfect the patient, we created a *coinfection exclusion score* (CES), see Supplementary material, Sect. 1.1, for the precise definition.

To exclude bias due to seasonal effects, we carried out this procedure for the entire data set stratified by months and averaged the resulting values over the statistically significant CES per virus pairs and months (Supplementary material, Sect. 1.2), yielding the *average coinfection exclusion score* (ACES). The CES and ACES scores assume a value of 1 if coinfections are ten times less likely than expected by chance and a value of -1 if they are ten times more likely.

## Results

### Database pre-processing

From 2010 to 2019, RespVir database registered 217,150 samples. After the initial filtering of the records with incomplete data, we retained 213,131 (98.14%) sample records.

To the monoinfection analysis, we selected samples with one or no positively tested pathogen. Regarding coinfections we selected all samples tested for at least two pathogens. To filter out sample records that are likely erroneous, for the coinfection analysis, we performed an additional post-hoc curation by setting a coinfection rate threshold of 0.01, for each of the virus pair per site. We discarded all coinfection patient records that yielded confections rates above this coinfection rate threshold.

After all filters were applied to the data base, we retained 206,814 sample records, of which 126,808 (61.31%) were monoinfections, 6781 (3.27%) coinfections, and 72,335 (34.97%) negatives. The majority of the samples, 26.69%, belonged to patients in ages between 0 < 6 years (26.69%), followed by patients between 45 < 65 years (23.96%) (Table 2). The retained samples correspond to 1,408,657 tests performed. For coinfection, we analysed 7,790,879 tests results combinations corresponding to the 197 senseful viral pairs (Supplementary table S1).

**Table 2:** Age distribution by age goup

Age group	# of samples	Percentage
0 < 6	55,199	26.69%
6 < 13	14,684	7.1%
13 < 19	10,382	5.02%
19 < 46	44,486	21.51%
46 < 65	49,553	23.96%
65 +	32,511	15.72%
Total	209,814	100%

Table 2 The table shows the age distribution and percentage of samples per age group of retained samples after filtering the database

### Infections prevalence

The 17 different respiratory viruses were not tested equally frequently. For example, while the 30.15% of the samples were tested only for the *Orthomyxoviridae* family, consisting of with FLUA (18.18%) and FLUB (11.97%). Members of the *Coronaviridae* family were tested significantly less frequently, with 4.51% HCoV-OC43, 4.36% HCoV-NL63, 4.21% HCoV-229E, 1.21% HCoV-HKU1, and 0.54% HCoV-generic (Table 1).

Influenza viruses represent 48.64% of all positive reports, of which 31.90% pertain to FLUA, and 16.74% to FLUB. The remaining 51.36% cover the remaining 14 viruses.

FLUA-generic test is the test with the highest positivity percentage (32.89%), followed by rhinovirus (28.23%), RV/EV (20.70%), human respiratory syncytial virus (HRSV) (20.37%), and influenza B (15.19%). The tests for coronaviruses and parainfluenza families were the ones with the lowest positivity percentage (Table 1).

### Seasonal variations of viruses circulation

**Fig. 2:**
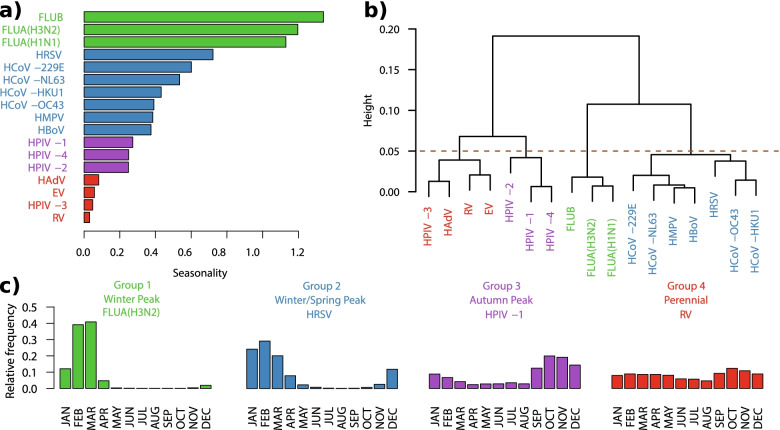
Seasonality Profile of the Respiratory Viruses. The figure shows the seasonality profile of the 17 respiratory viruses studied. **a**) Degree of seasonality of each virus calculated by Kullback–Leibler divergence, where zero indicates no seasonality (see Methods, seasonality profile). **b**) Average linkage clustering of the 17 viruses according to their seasonality profile. **c**) The seasonal four groups according to the similarities of the 17 viruses, the figure shows the seasonal profile of one virus per group and the group name

To study the seasonality of the respiratory viruses, we excluded the four tests that do not differentiate virus subtypes. The influenza viruses (FLUB, FLUA(H3N2), and FLUA(H1N1) exhibit the highest degree of seasonality, whereas RV, HPIV-3, enterovirus, and adenovirus exhibit the lowest (Fig. 2a).

We determined the similarity of the seasonality profiles among the 17 viruses and carried out hierarchical clustering with average linkage. After applying a cut-off of 0.05 to the dendrogram, we obtained four groups (Fig. 2b), each of which we assigned an interpretive label: (1) “Winter Peak” comprises FLUB, FLUA(H3N2) and FLUA(H1N1)), (2) “Winter/Spring Peak” comprises HRSV, HCoV-229E, HCoV-NL63, HCoV-HKU1, HCoV-OC43, HMPV, and HBoV, (3) “Autumn Peak” comprises HPIV-1, HPIV-2, and HPIV-4, and (4) “Perennial” comprises HAdV, EV, HPIV-3, RV (Fig. 2c and Supplementary figure S1). We repeated this analysis using the positivity percentage, obtaining similar results. (Supplementary figure S2).

**Fig. 3:**
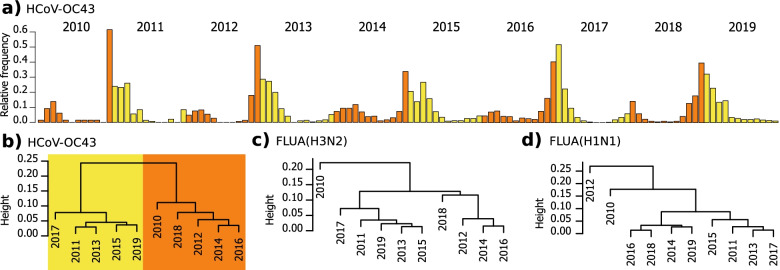
Annual variation of seasonality. The figure shows the annual variation of seasonality and the biennial pattern discovered. **a**) Biennial pattern of HCoV-OC43 exhibiting high infection numbers at the end of even and beginning of odd years, but low infection numbers at the end of odd and beginning of even years. **b**) Hierarchical clustering with average linkage of the annual variation of seasonality of HCoV-OC43. **c**) Hierarchical clustering with average linkage of the annual variation of seasonality of FLUA(H3N2). **d**) Hierarchical clustering with average linkage of the annual variation of seasonality of FLUA(H1N1)

To analyse seasonality variation of each virus among the 10 years of the study, we stratified the frequency of positive tests for each pathogen by year and calculated their year-specific seasonality profiles (Supplementary figure S3). We found that HCoV-OC43, exhibiting a clear biennial pattern, that is, high infection numbers at the end of even and beginning of odd years (Fig. 3a). To obtain a more concise representation, we applied hierarchical clustering with average linkage to the ten profiles of each virus (Supplementary figure S4). We found that the year-specific seasonality profiles of HCoV-OC43 resolve into two groups that contain the odd and even years respectively (Fig. 3b), and a similar biennial variation becomes apparent for HPIV-1, HPIV-3, and HRSV (Supplementary figure S4). For FLUA(H3N2) (Fig. 3c) and FLUA(H1N1) (Fig. 3d) a biennial pattern can be presumed, but it is not consistently true all years.

### Coinfections prevalence and virus pair relations

To describe the coinfection prevalence, we analysed 7,790,879 tests results for the 197 virus test pairs.

We found that RV/HBoV, RV/HAdV, HRSV/HBoV, HRSV/HAdV and HRSV/HCoV-OC43 coinfections were the most prevalent coinfections, and together with HRSV/RV had the highest positivity percentage. For 19 out of the 197 studied virus pairs, we did not find any coinfection case (Supplementary table S1).

**Fig. 4:**
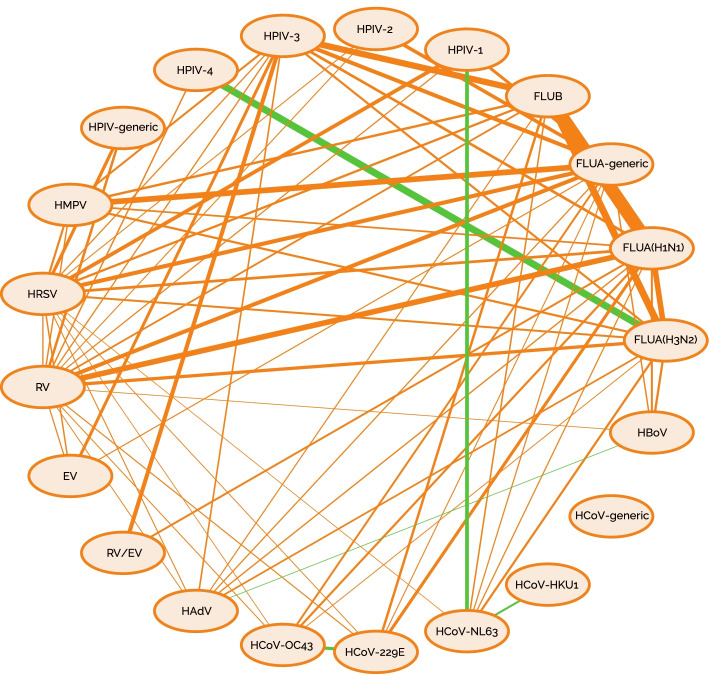
Interaction strength between the 17 virus pairs regarding coinfection. The figure shows the 17 studied viruses linked by lines. Orange lines indicate an exclusion interaction while green lines an affinity interaction. The thickness of the lines indicates the strength of the interaction

For 73 of the 197 virus pairs, we obtained a coinfection exclusion score (CES), which implies a statistically significant dependence of both test results, for at least one month. The great majority of test pairs yield a positive ACES (Average coinfection exclusion score), indicating coinfection exclusion. The pair FLUA(H1N1)/FLUB shows the highest coinfection exclusion (ACES = 1.67), followed by FLUA(H3N2)/FLUB (ACES = 1.39), FLUB/HPIV-3 (ACES = 1.34), FLUA-generic/HMPV (ACES = 1.30), and FLUA(H1N1)/RV and FLUA(H3N2)/FLUA(H1N1) with an ACES = 1.29 for both cases. We found negative ACES values in only five test pairs, indicating affinity of the viral pairs like FLUA(H3N2)/HPIV-4 with ACES = -1.39 (Fig. 4 and Supplementary table S2).

## Discussion

In the present study, we present data from the respiratory pathogens network that has been in place since 2009, based on multicenter, wide-spectrum collection rather than collection of data based on narrowly defined selection criteria. In principle it could be taken as a limitation due to the different clinical criteria by applied by physicians as they request specific diagnostic test. The multiplex test approach in our analysis reduces a possible bias because the samples are tested not only for a single suspected pathogen, but for the 17 respiratory viruses of the multiplex panel.

One disadvantage of this strategy is a lack of clinical historal data. For example, we cannot determine the influence of vaccination rate in our cohort. Nevertheless, the broad coverage (nationwide) of our data allows to assume that the vaccination rate is representative in our cohort.

Filtering and post-hoc curation was required. This was partly necessary due to non-curated data entering the database. To overcome this disadvantage, it is important to use quality control mechanisms during data collection in the future to reject the collection of false data mainly regarding coinfections.

We analysed the frequency of 17 respiratory pathogens with respect to monoinfections and coinfections, their seasonal variation, and the affinity to coinfect with other viruses, spanning 10 years (2010–2019). To our knowledge, this study reports on the largest volume of data of its kind [4, 8–15]. Nevertheless, another limitation in terms of global health is that our samples come mainly from Germany, Austria and Switzerland, these samples constitute a good basis to analyze the Central Europe (continental) region and therefor the the results about saisonality should not be extrapolated to other hemisphere regions, nor even to north Europe, Spain or United kingdom.

Our results confirm previous reports that influenza A viruses, HRSV, and RV were detected most frequently in our cohort [4, 13, 14, 16, 17]. Nevertheless, almost one quarter (24.65%) of the infections are caused by other respiratory viruses. Although influenza tests are the most frequently performed assays, 51.36% of all positive tests derived from other respiratory viruses. This supports the importance of testing for multiple pathogens for diagnostic purposes [18–20]. A disadvantage of our approach is that relies on routine diagnostic test, therefore influenza typing is restricted to Influenza A (H1N1 and H3N2) and no further data on the subtypes of influenza B are given, Consequently, no detailed specific description of the viruses' behaviour or more specific coinfection relations can be evaluated.

To detect patterns of seasonal variation worldwide, corresponding worldwide and long-term studies are needed [18, 19]. Our study is robust and covers a long-term period for the central European area. This study allows us to confirm previously reported seasonal patterns [18–22], but also to propose a new seasonal classification of the studied viruses into four groups. Furthermore, we found a typical seasonal pattern repeated every other years for HPIV-1, HPIV-3, HMPV, HRSV, and HCoV-OC43; also, for FLUA(H3N2) except for the years 2010 and 2018 as well as for FLUA(H1N1) except for years 2010 and 2012, respectively. This confirms the constancy of the biennial patterns except for years near to a pandemic event due to new viruses appearance.

The SARS-CoV-2 outbreak has raised questions regarding the seasonal pattern of this virus. Studies on seasonal patterns of endogenous viruses could help to solve these questions. We found a slight difference in the seasonal profile within the coronaviruses (Figure S1). SARS-CoV-2 belongs to beta-coronaviruses which season usually starts in November–December and has its peak in December-January. Thus, a similar seasonality could be expected for SARS-CoV-2 in the future.

Coinfection modifies the natural history of diseases caused by single infections. Thus, deeper understanding of coinfections, especially the exclusion mechanisms could help the development of antivirals [23]. Only a few large-scale data analyses on virus-virus interactions exist, in contrast to numerous studies on bacterial coinfections and virus-bacteria studies [24–28]. We characterized the coinfection prevalence and the interactions between 17 different viruses and analysed 7,790,879 tests combinations, within the ten year observation period. To our knowledge, our study provides the analysis of virus-virus interaction with the largest diversity of respiratory viruses, the longest surveillance period, and the largest number of tests performed.

As expected, the most prevalent coinfection virus pairs and the highest positivity percentage (Table S1) had also a high monoinfection prevalence and a seasonal overlap. To compare the propensity of a virus pair to coinfect, we introduced a coinfection exclusion score (CES). To exclude bias due to seasonal effects, we calculated an average coinfection exclusion score (ACES).

One of the most relevant studies of virus-virus interaction has been performed by Nickbakhsh et. al. [29], who analysed 44,230 respiratory illness cases tested for 11 viruses over nine years and classified the viral pairs interactions. Our data confirm a strong exclusion of any of the influenza A strains (H1N1 or H3N2) to coinfect with rhinovirus. This exclusion has been confirmed also in an animal model [30]. Our data also confirm an exclusion between FLUB and HAdV. In contrary to Nickbakhsh et al. [29], our data suggest strong exclusion for HRSV and HMPV coinfection and no significant interaction between HPIV-2 with HPIV-3. Nickbakhsh et al. [29] did not report any other interaction, while our data shows the strongest exclusion for FLUA(H1N1) and FLUB as well as for FLUA(H3N2) and FLUB. For the other virus pairs the numbers are too small to test for significance. So, further studies are needed to get more insight into the frequency ant role of virus co-infections.

## Conclusions

The deeper understanding on virus dynamics will contribute to improve diagnostics, the prevention of infection, and potentially, the development of therapies for viral infections. We show the advantages of multiplex testing to identify the causative agent for a respiratory disease. Our approach shows the usefulness for collecting data with such real world data bases. Analysis of data on (co-) infections, seasonality, and interactions of viruses can be performed much faster compared to prospective clinical studies.

## Supplementary Information

**Additional file 1. Supplementary file 1.** 

## Data Availability

Data and source code are available upon reasonable request from Rolf Kaiser and Gibran Horemheb-Rubio (data) and Ralf Eggeling (source code).

## Abbreviations

RespVir: Respiratory Viruses Network
ACES: Average coinfection exclusion score
COVID-19: Coronavirus disease 2019
SARS-CoV-2: Severe acute respiratory disease-coronavirus-2
GfV: German Virology Society
FLUA: Generic: influenza A generic test
HPIV: Generic: human parainfluenza virus-generic test
HCoV: Human coronavirus
HCoV-OC43: Human coronavirus OC43
HCoV-NL63: Human coronavirus NL63
HCoV-229E: Human coronavirus 229E
HCoV-HKU1: Human coronavirus HKU1
RV/EV: Rhinovirus/enterovirus
KLD: Kullback-Leibler divergence
CES: Coinfection exclusion score
FLUA: Influenza A
FLUB: Influenza B
HRSV: Human respiratory syncytial virus
HMPV: Human metapneumovirus
HBoV: Human bocavirus
HPIV-1: Human parainfluenza virus 1
HPIV-2: Human parainfluenza virus 2
HPIV-4: Human parainfluenza virus 4
HAdV: Human adenovirus
EV: Enterovirus
HPIV-3: Human parainfluenza virus 3
RV: Rhinovirus
