# Supplementary material for: Respiratory viruses dynamics and interactions: ten years of surveillance in central Europe
Source: BMC Public Health. 2022 Jun 11;22:1167. doi: 10.1186/s12889-022-13555-5 (PMC9187845; doi:10.1186/s12889-022-13555-5)
Supplement: Supplementary file 1 — Additional file 1. Supplementary file 1. [file 12889_2022_13555_MOESM1_ESM.pdf]

Respiratory Viruses Dynamics and Interactions: Ten Years of Surveillance in Central Europe

Additional File 1 - Supplementary Material

Gibran Horemheb-Rubio, PhD[*1,2], Ralf Eggeling, PhD[*3], Norbert Schmeisser, Dr. rer. medic[4], Nico Pfeifer, Dr. rer. Nat.[3,5,6,7], Thomas Lengauer, PhD[1,7], Barbara C. Gärtner, MD[8], Christiane Prifert, M.sc.[9], Matthias Kochanek, MD[10], Christoph Scheid[10], MD and Ortwin Adams, MD[11**], Rolf Kaiser, PhD[1**]. On behalf of the Respiratory Virus Network

*Contributed equally

**Also contributed equally

[1]Institute of Virology, Faculty of Medicine and University Hospital Cologne, Fürst-Pückler-Straße 56, 50935 Cologne, Germany, [2]Department of Infectious Diseases, Instituto Nacional de Ciencias Médicas y Nutrición Salvador Zubirán, Vasco de Quiroga 15, 14080 Mexico City, Mexico, [3]Methods in Medical Informatics, Department of Computer Science, University of Tübingen, Sand 14, 72076 Tübingen, Germany, [4]Medeora GmbH, Am Gleisdreieck 1, 50823 Cologne, Germany, [5]Faculty of Medicine, University of Tübingen, 72076 Tübingen, Germany, [6]German Center for Infection Research, Partner Site Tübingen, 72076 Tübingen, Germany, [7]Computational Biology, Max Planck Institute for Informatics, Saarland Informatics Campus, 66123 Saarbrücken, Germany, [8]Institute of Medicine Microbiology and Hygiene, University of the Saarland Kirrberger- Straße Building 43, 66421 Homburg/Saar, [9]Faculty of Medicine, Institute for Virology and Immunobiology, Würzburg University, Sanderring 2, 97070 Würzburg, Germany, [10]University of Cologne, Department of Internal Medicine, Center for Integrated Oncology Aachen Bonn Cologne Düsseldorf, Kerpener Straße 62, 50937 Cologne, Germany, [11]University of Düsseldorf, Medical Faculty, Institute for Virology, 40225 Düsseldorf, Germany

Table of contents

1.- Methods

1.1 Definition of the coinfection exclusion score

1.2 Definition of the average coinfection exclusion score (ACES)

2.- Figure S1

3.- Figure S2

4.- Figure S3

5.- Figure S4

6.- Table S1

7.- Table S2

1. Methods

1.1 Definition of the coinfection exclusion score

Let $X_i \in \{0,1\}$ denote a binary random variable that assume a value of 1 if the test result for pathogen *i* is positive. To assess the rate of coinfection among pathogens *i* and *j* in a given time interval, we apply the following procedure. First, we consider only patients for whom a result (positive or negative) is available for both *i* and *j*.

We carry out a chi-square test to assess the statistical independence of X_i and X_j. We use a significance threshold α=0.05 and Bonferroni correction.

For all cases in which statistical independence is rejected, we compute $p_{ij} = \hat{P}(X_i = 1, X_j = 1)$, the empirical joint probability of a positive result for both tests. We further compute $p_i = \hat{P}(X_i = 1)$ and $p_j = \hat{P}(X_j = 1)$, the empirical marginal probabilities of a positive result for each of the two tests. The product $p_i \cdot p_j$ quantifies the expected coinfection frequency under the assumption that both pathogens infect independently.

Finally, we define the *coinfection exclusion score* (CES) as

$$\mathrm{CES}_{ij} = -\log_{10}\left(\frac{p_{ij}}{p_i \cdot p_j}\right)$$

The score assumes a value of 1 if coinfections are ten times less likely than expected by chance, and a value of -1 if they are ten times as likely as expected by chance.

1.2 Definition of the average coinfection exclusion score (ACES)

In order to exclude bias due to seasonal effects that may create artificially high or low coinfection frequencies, we calculate the CES for the entire datasets stratified by months. As a consequence, the Bonferroni correction is based on a number of 12.197=2364 chi-square tests that are carried out. Subsequently we compute weighted average of the resulting CES values for each virus pair with the following quantity that we call the *average coinfection exclusion score* (ACES)

$$\mathrm{ACES}_{ij} = \frac{\sum_{m=1}^{12} \mathrm{CES}_{ij}^m \cdot s_{ij}^m \cdot N_{ij}^m}{\sum_{m=1}^{12} s_{ij}^m \cdot N_{ij}^m}$$

Here CES_{ij}^m denotes the coinfection exclusion score of the *m*-th month, N_{ij}^m denotes the total number of patients with test results for pathogens *i* and *j* in month *m*, and

$$s_{ij}^m = \begin{cases} 0, & \text{if } \mathrm{CES}_{ij}^m \text{ undefined or } \mathrm{CES}_{ij}^m = \infty \\ 1, & \text{otherwise} \end{cases}$$

is a selection variable which excludes all months for which statistical independence was not rejected (indicated by an undefined CES) or where at least one pathogen has zero positive tests (infinite CES).

Figure S1. Groups by seasonality profiles by the total number of positives

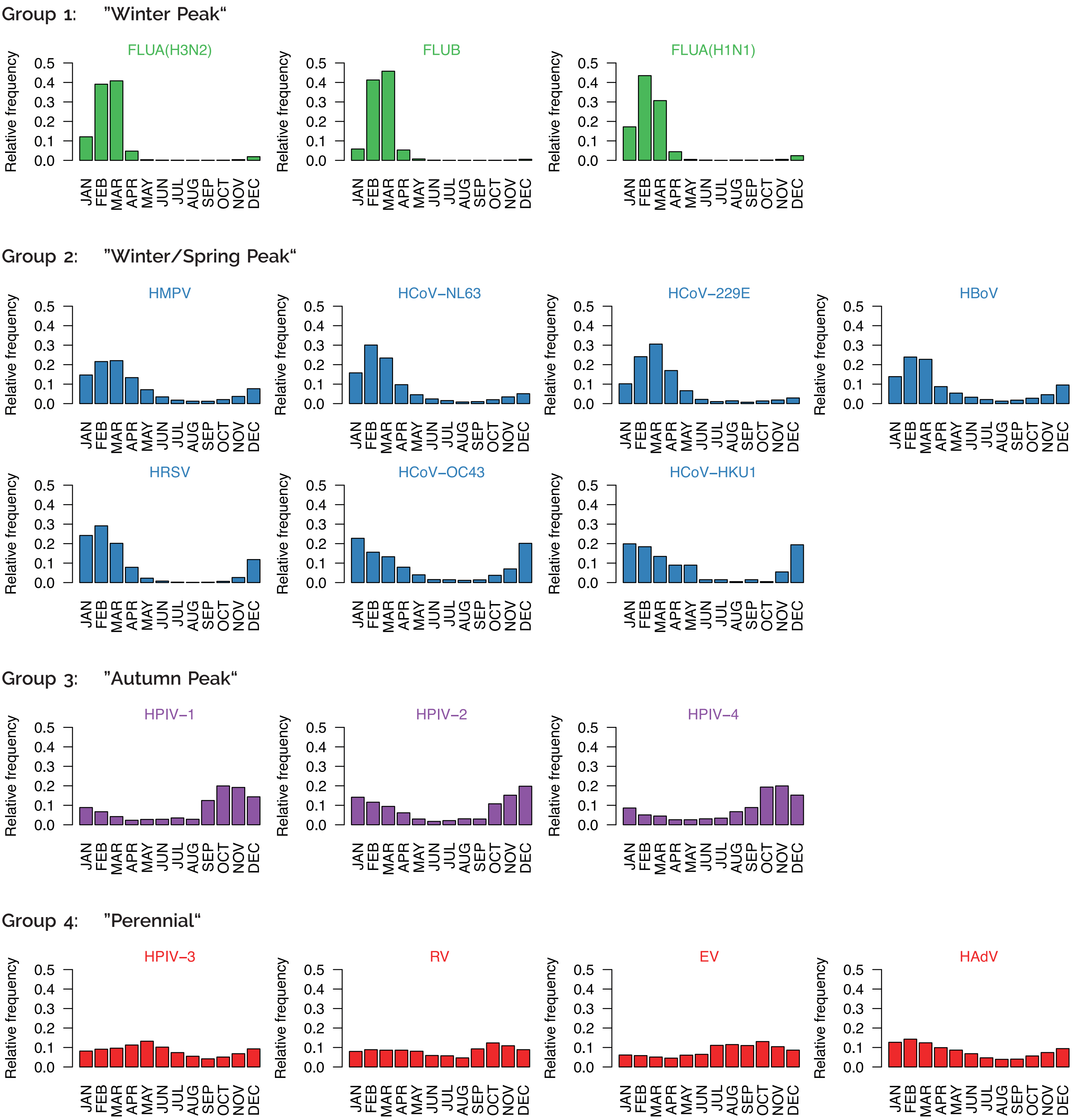

Figure S1. Groups by seasonality profiles by the total number of positives. The figure shows the four seasonal groups according to the similarities of the 17 viruses, calculated by the total number of positives.

Figure S2. Groups by seasonality profiles by the positivity rate

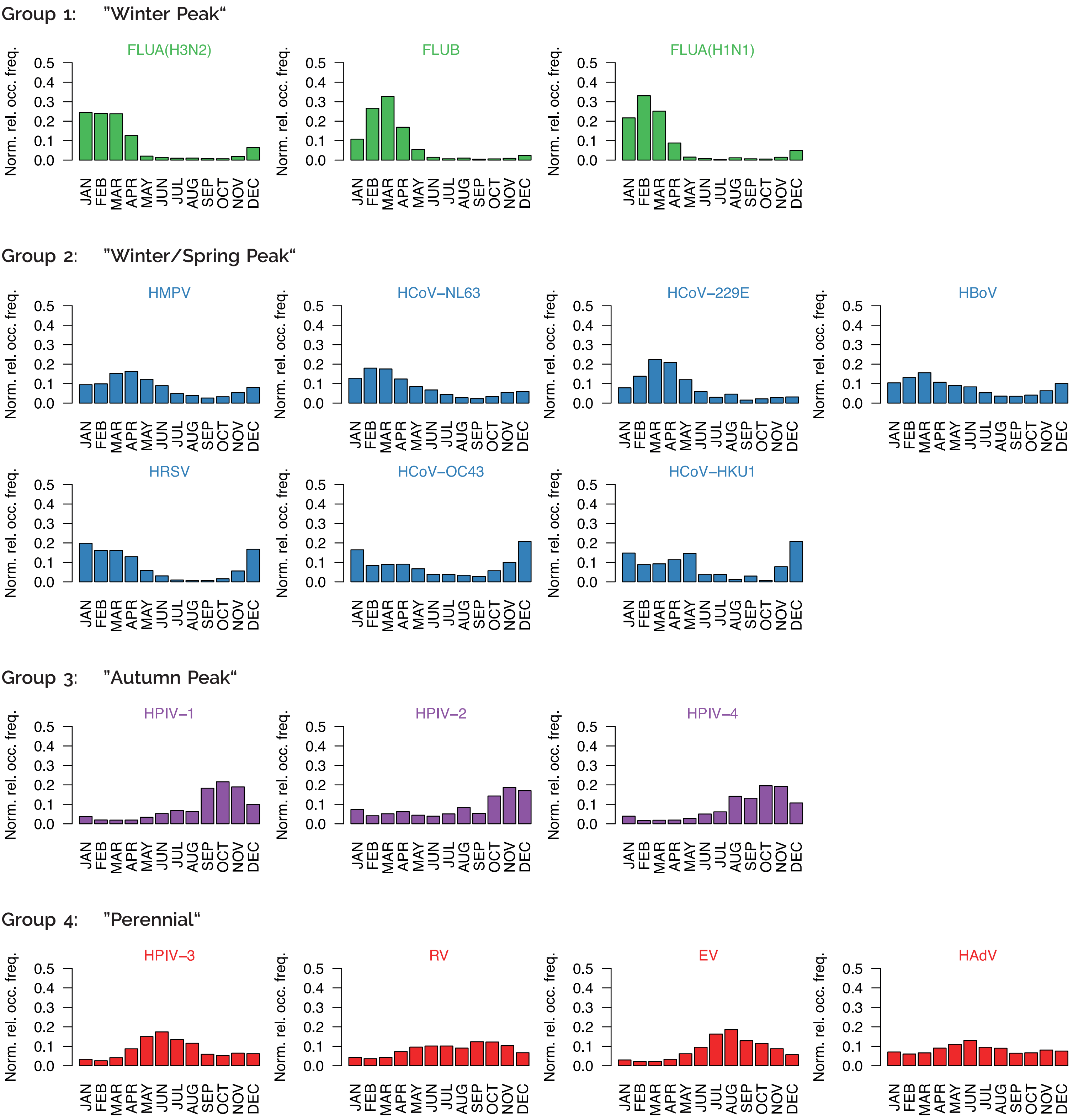

Figure S2. Groups by seasonality profiles by the positivity rate. The figure shows the seasonal four groups according to the similarities of the 17 viruses, calculated by the positivity rate.

Figure S3. Year-specific seasonality profiles

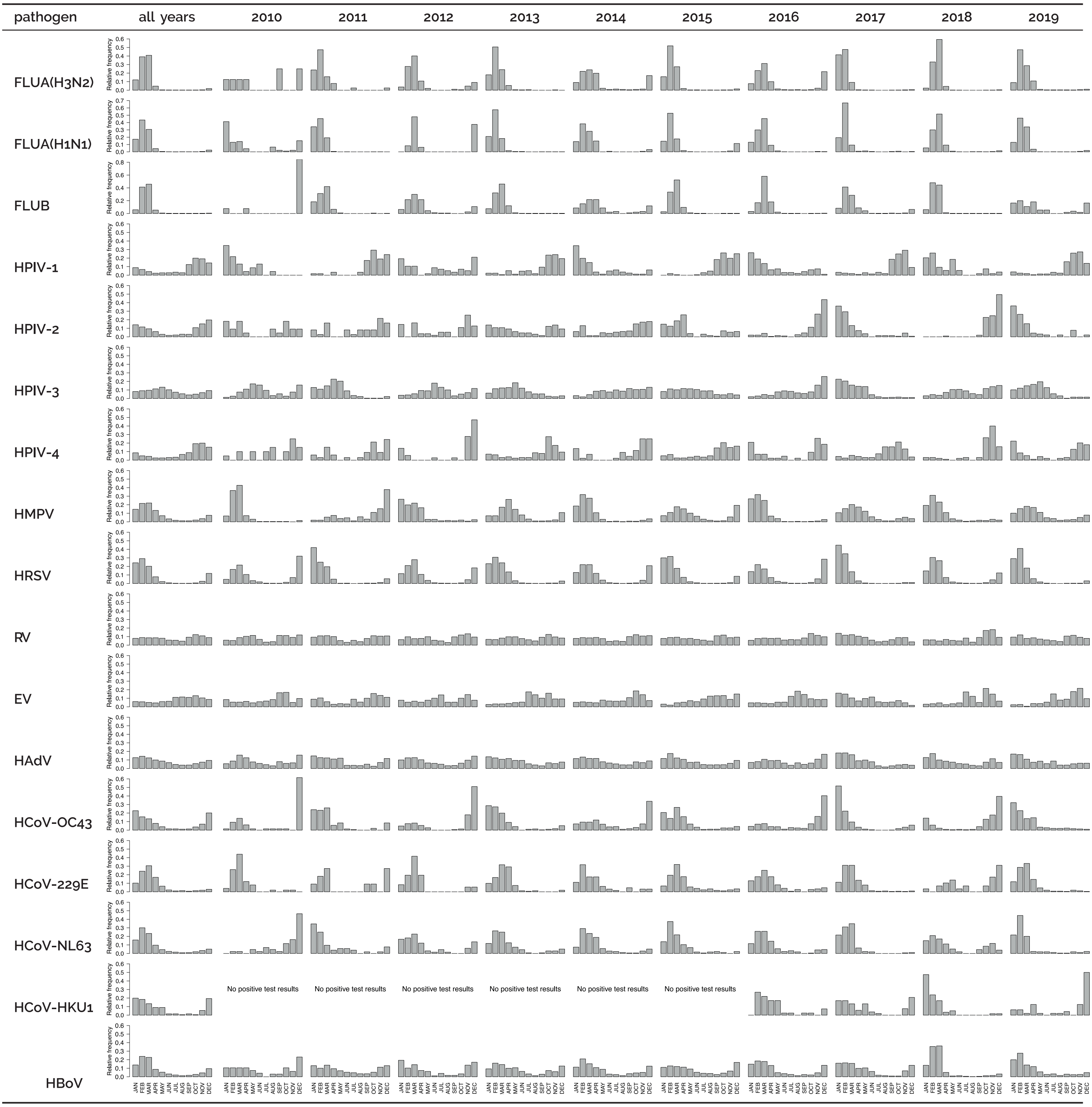

Figure S3. Year-specific seasonality profiles. The figure shoes the stratified frequency of positive tests for each pathogen by year.

Figure S4. Hierarchical clustering of virus seasonality profiles per year

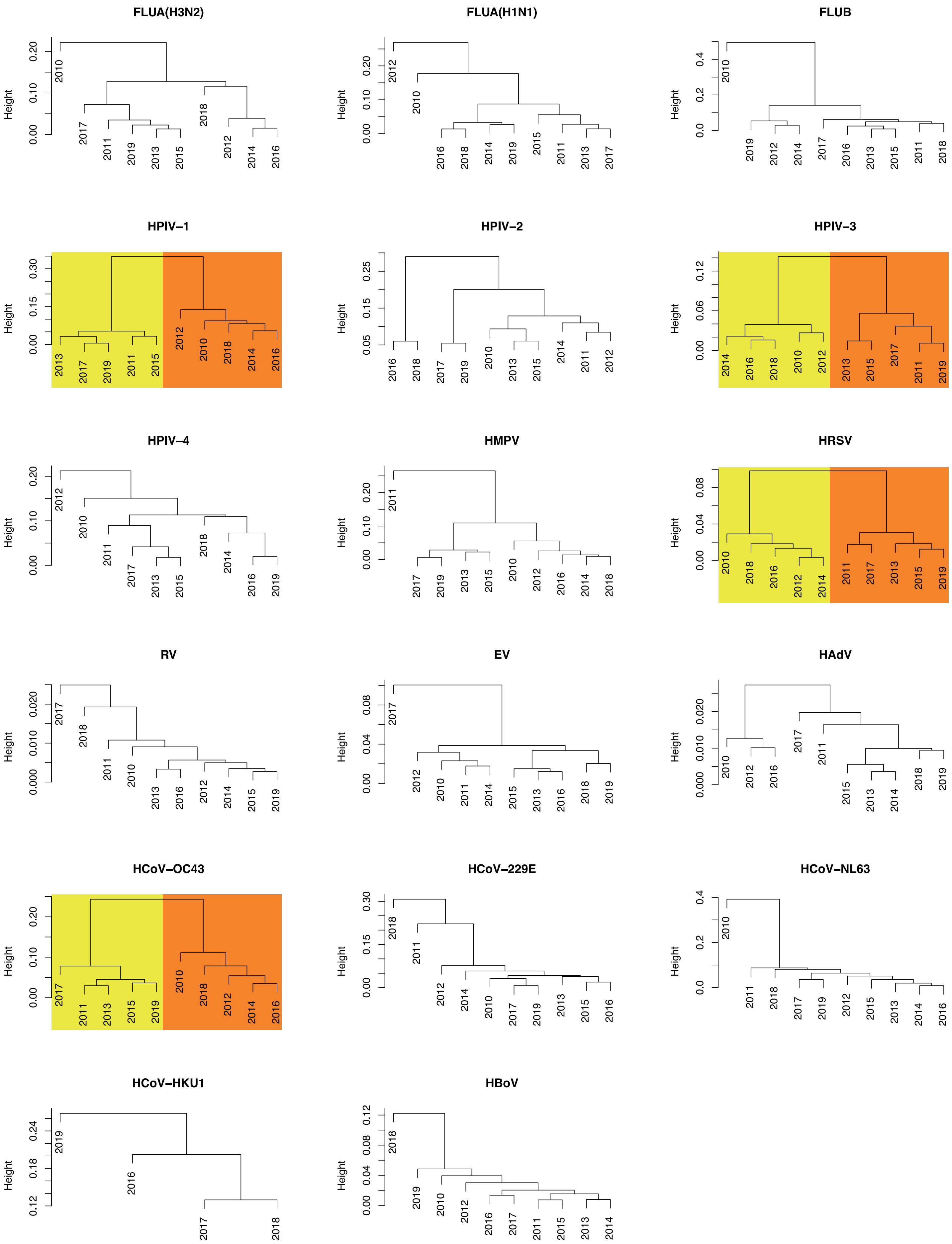

Figure S4. Hierarchical clustering of virus seasonality profiles per year. The figure shows the hierarchical clustering with average linkage to the ten profiles of each virus. The viruses that exhibit a biennial pattern are colored in orange and yellow.

6. Table S1. Coinfection Prevalence of Respiratory Viruses

Coinfection Prevalence of Respiratory Viruses								
Virus 1	Virus 2	Negatives	Monoinfection Virus 1	Monoinfection Virus 2	Coinfection	Total Tests	Positivity Rate	Coinfection Frequency
RV	HBoV	41,529	11,888	1,984	312	55,713	0.560%	4.6%
RV	HAdV	34,286	9,717	1,957	281	46,241	0.608%	4.2%
HRSV	HBoV	46,230	8,620	2,145	273	57,268	0.477%	4.0%
HRSV	HAdV	53,789	12,072	4,748	267	70,876	0.377%	3.9%
HRSV	HCoV-OC43	48,916	9,772	1,982	226	60,896	0.371%	3.3%
HPIV-3	RV	42,973	3,008	14,326	222	60,529	0.367%	3.3%
HAdV	HBoV	54,103	3,931	2,299	194	60,527	0.321%	2.9%
HRSV	RV	17,425	4,509	6,747	183	28,864	0.634%	2.7%
HMPV	RV	43,660	3,033	14,602	162	61,457	0.264%	2.4%
RV	EV	33,397	8,033	1,260	154	42,844	0.359%	2.3%
FLUA-generic	HRSV	34,664	10,971	7,680	154	53,469	0.288%	2.3%
HMPV	HAdV	67,098	4,020	5,055	153	76,326	0.200%	2.3%
RV	HCoV-OC43	41,381	12,879	1,857	150	56,267	0.267%	2.2%
HRSV	HCoV-NL63	49,981	9,737	1,150	135	61,003	0.221%	2.0%
FLUA(H3N2)	HRSV	32,247	3,279	7,565	131	43,222	0.303%	1.9%
FLUB	HRSV	61,309	4,490	14,106	123	80,028	0.154%	1.8%
HMPV	HRSV	65,281	4,022	13,276	113	82,692	0.137%	1.7%
HMPV	HBoV	56,802	3,229	2,511	112	62,654	0.179%	1.7%
HPIV-3	HRSV	60,927	3,423	12,076	111	76,537	0.145%	1.6%
HRSV	HCoV-229E	48,078	9,785	875	106	58,844	0.180%	1.6%
HPIV-3	HAdV	60,185	3,458	4,910	105	68,658	0.153%	1.6%
FLUA(H3N2)	FLUB	60,762	9,918	15,976	103	86,759	0.119%	1.5%
FLUB	RV	40,365	2,542	15,275	79	58,261	0.136%	1.2%
EV	HAdV	53,283	2,874	3,320	79	59,556	0.133%	1.2%
HRSV	EV	46,587	9,685	3,080	78	59,430	0.131%	1.2%
HAdV	HCoV-OC43	56,251	4,321	2,210	77	62,859	0.122%	1.1%
RV	HCoV-NL63	41,510	12,823	1,094	75	55,502	0.135%	1.1%
HPIV-1	RV	45,266	711	14,422	74	60,473	0.122%	1.1%
FLUB	HAdV	59,014	3,072	5,062	73	67,221	0.109%	1.1%
HPIV-3	HBoV	51,872	2,846	2,923	65	57,706	0.113%	1.0%
FLUA(H1N1)	HRSV	38,890	2,214	8,896	64	50,064	0.128%	0.9%
RV	HCoV-229E	39,598	13,037	880	59	53,574	0.110%	0.9%
HPIV-3	HCoV-OC43	57,152	3,229	2,153	59	62,593	0.094%	0.9%

HPIV-2	RV	45,057	629	14,234	58	59,978	0.097%	0.9%
HCoV-OC43	HBoV	52,232	1,992	2,923	57	57,204	0.100%	0.8%
EV	HBoV	50,848	3,124	2,773	56	56,801	0.099%	0.8%
HPIV-4	RV	29,007	471	9,770	55	39,303	0.140%	0.8%
HAdV	HCoV-NL63	55,534	4,203	1,230	55	61,022	0.090%	0.8%
FLUA-generic	HAdV	32,933	3,580	2,188	51	38,752	0.132%	0.8%
FLUA(H3N2)	HCoV-OC43	34,527	3,070	1,354	46	38,997	0.118%	0.7%
FLUA(H3N2)	HBoV	36,425	3,030	3,230	44	42,729	0.103%	0.7%
FLUB	HBoV	47,018	2,459	2,380	44	51,901	0.085%	0.7%
HPIV-3	EV	51,523	3,100	3,365	41	58,029	0.071%	0.6%
HMPV	HCoV-OC43	57,345	3,354	2,254	40	62,993	0.063%	0.6%
FLUA(H3N2)	RV	26,052	2,599	8,959	38	37,648	0.101%	0.6%
FLUA(H3N2)	HAdV	38,889	3,328	3,136	36	45,389	0.079%	0.5%
HPIV-2	HRSV	62,990	790	12,092	36	75,908	0.047%	0.5%
FLUA(H1N1)	HAdV	46,368	2,274	3,693	34	52,369	0.065%	0.5%
HCoV-NL63	HBoV	52,803	1,163	2,458	34	56,458	0.060%	0.5%
FLUB	HMPV	65,708	3,786	3,832	32	73,358	0.044%	0.5%
HPIV-3	RV/EV	6,504	306	1,721	31	8,562	0.362%	0.5%
FLUA(H3N2)	HMPV	39,854	3,354	2,499	30	45,737	0.066%	0.4%
EV	HCoV-OC43	51,141	3,355	2,003	29	56,528	0.051%	0.4%
HMPV	EV	55,321	3,130	3,152	29	61,632	0.047%	0.4%
HPIV-3	HMPV	67,857	3,454	3,593	29	74,933	0.039%	0.4%
FLUA(H3N2)	FLUA(H1N1)	43,265	3,852	2,881	28	50,026	0.056%	0.4%
FLUA(H1N1)	HMPV	47,297	2,269	2,927	27	52,520	0.051%	0.4%
HPIV-3	HCoV-229E	54,586	3,077	949	27	58,639	0.046%	0.4%
HAdV	HCoV-229E	53,658	4,260	977	27	58,922	0.046%	0.4%
HPIV-generic	RV	9,126	473	2,853	26	12,478	0.208%	0.4%
FLUA-generic	HMPV	35,844	9,467	2,137	26	47,474	0.055%	0.4%
HMPV	HCoV-NL63	56,649	3,195	1,262	26	61,132	0.043%	0.4%
HPIV-1	HAdV	62,760	921	4,972	25	68,678	0.036%	0.4%
FLUA-generic	RV	22,726	2,768	7,176	24	32,694	0.073%	0.4%
HCoV-OC43	HCoV-229E	55,805	2,149	955	24	58,933	0.041%	0.4%
FLUA-generic	HBoV	26,974	2,467	1,110	21	30,572	0.069%	0.3%
FLUA(H3N2)	HCoV-229E	31,836	3,019	571	21	35,447	0.059%	0.3%
HMPV	HCoV-229E	54,841	3,174	989	21	59,025	0.036%	0.3%
FLUB	HCoV-OC43	49,569	2,557	2,079	20	54,225	0.037%	0.3%
HPIV-2	HAdV	62,399	801	4,911	20	68,131	0.029%	0.3%
HRSV	RV/EV	4,083	388	561	19	5,051	0.376%	0.3%
FLUB	RV/EV	6,309	503	1,742	19	8,573	0.222%	0.3%
HPIV-1	HBoV	53,934	670	2,970	19	57,593	0.033%	0.3%

HPIV-3	HCoV-NL63	56,224	3,112	1,249	19	60,604	0.031%	0.3%
FLUA-generic	EV	24,341	2,385	1,772	18	28,516	0.063%	0.3%
FLUA(H3N2)	HPIV-3	36,105	3,293	2,099	18	41,515	0.043%	0.3%
FLUA(H1N1)	HBoV	44,006	1,918	2,130	18	48,072	0.037%	0.3%
HRSV	HCoV-generic	6,126	752	524	17	7,419	0.229%	0.3%
FLUA(H1N1)	HCoV-OC43	42,369	2,026	1,747	17	46,159	0.037%	0.3%
FLUB	HCoV-NL63	48,554	2,489	1,148	17	52,208	0.033%	0.3%
HPIV-2	HBoV	53,397	662	2,967	17	57,043	0.030%	0.3%
FLUA-generic	HCoV-OC43	28,820	2,492	1,179	16	32,507	0.049%	0.2%
HPIV-4	EV	35,675	502	2,768	16	38,961	0.041%	0.2%
FLUA-generic	HPIV-3	32,711	9,311	1,674	16	43,712	0.037%	0.2%
HPIV-4	HAdV	41,441	606	3,153	16	45,216	0.035%	0.2%
FLUA(H1N1)	RV	33,256	1,754	11,315	16	46,341	0.035%	0.2%
HCoV-OC43	HCoV-NL63	57,623	2,126	1,252	16	61,017	0.026%	0.2%
HPIV-1	RV/EV	6,619	97	1,735	15	8,466	0.177%	0.2%
FLUA-generic	HCoV-229E	26,094	2,469	518	15	29,096	0.052%	0.2%
FLUA-generic	HCoV-NL63	28,167	2,366	589	15	31,137	0.048%	0.2%
FLUA(H1N1)	HCoV-NL63	42,421	1,993	1,059	15	45,488	0.033%	0.2%
HPIV-4	RV/EV	6,714	93	1,738	14	8,559	0.164%	0.2%
HPIV-generic	HRSV	10,695	782	2,803	14	14,294	0.098%	0.2%
FLUA(H3N2)	HCoV-NL63	34,467	3,080	829	14	38,390	0.036%	0.2%
HPIV-4	HRSV	37,738	585	7,008	14	45,345	0.031%	0.2%
HPIV-1	EV	53,767	759	3,385	14	57,925	0.024%	0.2%
HCoV-229E	HCoV-NL63	55,971	948	1,231	14	58,164	0.024%	0.2%
HPIV-2	HCoV-OC43	59,550	742	2,190	14	62,496	0.022%	0.2%
HPIV-2	HMPV	69,852	796	3,600	14	74,262	0.019%	0.2%
HPIV-1	HMPV	70,276	917	3,604	14	74,811	0.019%	0.2%
FLUB	HCoV-229E	46,866	2,378	888	13	50,145	0.026%	0.2%
RV/EV	HAdV	4,382	557	89	12	5,040	0.238%	0.2%
RV/EV	HCoV-OC43	6,191	1,552	194	12	7,949	0.151%	0.2%
EV	HCoV-NL63	50,221	3,335	1,139	12	54,707	0.022%	0.2%
HPIV-1	HCoV-NL63	58,670	732	1,253	12	60,667	0.020%	0.2%
HPIV-1	HRSV	63,351	911	12,153	12	76,427	0.016%	0.2%
HCoV-HKU1	HBoV	14,194	135	1,070	11	15,410	0.071%	0.2%
HCoV-NL63	HCoV-HKU1	16,504	295	125	11	16,935	0.065%	0.2%
HPIV-4	HMPV	42,336	610	2,541	11	45,498	0.024%	0.2%
FLUB	EV	45,694	2,195	3,294	11	51,194	0.021%	0.2%
FLUA-generic	HCoV-generic	6,404	413	452	10	7,279	0.137%	0.1%
RV/EV	HCoV-NL63	6,255	1,554	130	10	7,949	0.126%	0.1%
FLUA-generic	HPIV-generic	11,372	947	586	10	12,915	0.077%	0.1%

HMPV	HCoV-HKU1	15877	867	146	10	16,900	0.059%	0.1%
HPIV-4	HBoV	36,680	492	2,159	10	39,341	0.025%	0.1%
FLUA(H3N2)	HPIV-2	37,024	3,353	515	10	40,902	0.024%	0.1%
HPIV-2	EV	53,836	667	3,390	10	57,903	0.017%	0.1%
FLUA(H3N2)	RV/EV	5,220	246	1,297	9	6,772	0.133%	0.1%
HRSV	HCoV-HKU1	14,654	1,970	127	9	16,760	0.054%	0.1%
FLUA(H1N1)	HPIV-3	43,277	2,218	2,657	9	48,161	0.019%	0.1%
EV	HCoV-229E	48,402	3,371	921	9	52,703	0.017%	0.1%
FLUA(H1N1)	FLUB	52,444	3,619	4,372	9	60,444	0.015%	0.1%
HPIV-2	HPIV-3	71,873	812	3,528	9	76,222	0.012%	0.1%
HPIV-generic	HCoV-generic	6,586	144	450	8	7,188	0.111%	0.1%
FLUA(H3N2)	HPIV-4	25,332	2,359	385	8	28,084	0.028%	0.1%
FLUA(H1N1)	HCoV-229E	39,845	1,914	803	8	42,570	0.019%	0.1%
HPIV-2	HPIV-4	43,804	519	579	8	44,910	0.018%	0.1%
HAdV	HCoV-HKU1	15,226	1,404	147	7	16,784	0.042%	0.1%
FLUA(H3N2)	EV	34,325	2,977	2,071	7	39,380	0.018%	0.1%
HPIV-1	HPIV-4	44,274	606	597	7	45,484	0.015%	0.1%
HPIV-3	HPIV-4	42,489	2,481	596	7	45,573	0.015%	0.1%
FLUB	HPIV-3	60,763	3,778	3,241	7	67,789	0.010%	0.1%
HPIV-1	HPIV-3	72,299	919	3,532	7	76,757	0.009%	0.1%
RV/EV	HCoV-229E	3,429	1,512	60	6	5,007	0.120%	0.1%
HMPV	RV/EV	4,321	154	581	6	5,062	0.119%	0.1%
FLUA(H1N1)	RV/EV	5,033	435	1,302	6	6,776	0.089%	0.1%
FLUB	HCoV-generic	6,405	471	532	6	7,414	0.081%	0.1%
HMPV	HCoV-generic	6,628	281	535	6	7,450	0.081%	0.1%
RV/EV	HCoV-HKU1	6,355	1,556	29	6	7,946	0.076%	0.1%
FLUB	HPIV-generic	16,926	2,326	826	6	20,084	0.030%	0.1%
HPIV-4	HCoV-229E	38,345	499	726	6	39,576	0.015%	0.1%
HPIV-4	HCoV-OC43	41,369	558	1,590	6	43,523	0.014%	0.1%
FLUA(H1N1)	HPIV-2	44,682	2,179	623	6	47,490	0.013%	0.1%
RV	HCoV-HKU1	8,871	2,630	116	5	11,622	0.043%	0.1%
FLUB	HCoV-HKU1	15,666	887	127	5	16,685	0.030%	0.1%
FLUA-generic	HPIV-2	33,836	9,320	450	5	43,611	0.011%	0.1%
HCoV-229E	HBoV	50,451	935	2,937	5	54,328	0.009%	0.1%
HPIV-2	HCoV-NL63	58,688	715	1,261	5	60,669	0.008%	0.1%
HPIV-1	HCoV-OC43	59,496	794	2,203	5	62,498	0.008%	0.1%
HAdV	HCoV-generic	6,591	287	537	4	7,419	0.054%	0.1%
FLUA(H1N1)	HPIV-4	33,349	1,906	508	4	35,767	0.011%	0.1%
HPIV-generic	RV/EV	3,313	16	245	3	3,577	0.084%	0.0%
HCoV-generic	HBoV	5,338	272	160	3	5,773	0.052%	0.0%

HPIV-3	HCoV-generic	5,784	220	336	3	6,343	0.047%	0.0%
HPIV-2	RV/EV	6,605	104	1,747	3	8,459	0.035%	0.0%
HPIV-generic	HAdV	12,336	547	647	3	13,533	0.022%	0.0%
HPIV-generic	HMPV	12,414	740	698	3	13,855	0.022%	0.0%
HPIV-2	HCoV-229E	56,958	717	972	3	58,650	0.005%	0.0%
HPIV-2	HCoV-HKU1	16,479	194	132	2	16,807	0.012%	0.0%
HPIV-4	HCoV-NL63	41,200	511	872	2	42,585	0.005%	0.0%
FLUA-generic	HPIV-1	33,827	9,326	458	2	43,613	0.005%	0.0%
FLUA(H1N1)	EV	42,343	1,771	2,445	2	46,561	0.004%	0.0%
HPIV-1	HCoV-229E	56,935	731	972	2	58,640	0.003%	0.0%
FLUB	HPIV-2	62,609	3,776	770	2	67,157	0.003%	0.0%
FLUB	HPIV-1	63,087	3,780	824	2	67,693	0.003%	0.0%
RV	HCoV-generic	4,089	601	87	1	4,778	0.021%	0.0%
HPIV-generic	HCoV-NL63	6,934	134	76	1	7,145	0.014%	0.0%
FLUA-generic	RV/EV	6,064	283	1,430	1	7,778	0.013%	0.0%
HPIV-generic	HBoV	7,888	226	250	1	8,365	0.012%	0.0%
FLUA-generic	HCoV-HKU1	10,554	544	92	1	11,191	0.009%	0.0%
FLUA(H1N1)	HCoV-HKU1	11,227	790	110	1	12,128	0.008%	0.0%
FLUA(H3N2)	HCoV-HKU1	11,583	669	115	1	12,368	0.008%	0.0%
HPIV-4	HCoV-HKU1	16,414	178	134	1	16,727	0.006%	0.0%
HPIV-1	HCoV-HKU1	16,498	183	133	1	16,815	0.006%	0.0%
HPIV-3	HCoV-HKU1	16,049	725	133	1	16,908	0.006%	0.0%
FLUB	HPIV-4	40,956	1,964	589	1	43,510	0.002%	0.0%
FLUA(H1N1)	HPIV-1	45,138	2,225	683	1	48,047	0.002%	0.0%
HPIV-1	HPIV-2	74,498	916	819	1	76,234	0.001%	0.0%
FLUA(H3N2)	HPIV-1	37,515	3,366	576	0	41,457	0.000%	0.0%
FLUA(H3N2)	HPIV-generic	11,207	1,171	178	0	12,556	0.000%	0.0%
FLUA(H3N2)	HCoV-generic	4,171	472	80	0	4,723	0.000%	0.0%
FLUA(H1N1)	HPIV-generic	11,290	1,089	185	0	12,564	0.000%	0.0%
FLUA(H1N1)	HCoV-generic	4,355	283	79	0	4,717	0.000%	0.0%
FLUA-generic	HPIV-4	23,298	2,305	349	0	25,952	0.000%	0.0%
HPIV-1	HCoV-generic	5,835	75	339	0	6,249	0.000%	0.0%
HPIV-2	HCoV-generic	5,862	41	339	0	6,242	0.000%	0.0%
HPIV-4	HCoV-generic	5,948	57	338	0	6,343	0.000%	0.0%
HPIV-generic	EV	7,832	165	158	0	8,155	0.000%	0.0%
HPIV-generic	HCoV-OC43	6,815	140	219	0	7,174	0.000%	0.0%
HPIV-generic	HCoV-229E	3,999	135	70	0	4,204	0.000%	0.0%
HPIV-generic	HCoV-HKU1	3,144	21	5	0	3,170	0.000%	0.0%
EV	HCoV-HKU1	9,563	1,127	110	0	10,800	0.000%	0.0%
EV	HCoV-generic	5,379	86	248	0	5,713	0.000%	0.0%

RV/EV	HCoV-generic	2,942	61	1	0	3,004	0.000%	0.0%
RV/EV	HBoV	2,998	80	38	0	3,116	0.000%	0.0%
HCoV-OC43	HCoV-HKU1	16,297	500	136	0	16,933	0.000%	0.0%
HCoV-229E	HCoV-HKU1	13,548	239	131	0	13,918	0.000%	0.0%
**Total**		**6,811,616**	**484,095**	**488,407**	**6,761**	**7,790,879**		**100%**

Table S1. Coinfection Prevalence of Respiratory Viruses. The table shows the 197 valid virus pairs for the coinfection study. Each row pertains to a specific coinfecting virus pair and shows the name of the viruses, the total number of negative outcomes for both viruses, the total number of positives outcomes for each virus, the total number of positive outcomes for both viruses and the total number of tests performed for each virus pair. The table also shows the positivity rate of each coinfection and the frequency of this coinfection among all coinfections.

7. **Table S2. Coinfection Interactions**

Average Exclusion Score (ACES)														
		Coinfection Exclusion Score (CES) for every month among 10 years												
**Pathogen 1**	**Pathogen 2**	**JAN**	**FEB**	**MAR**	**APR**	**MAY**	**JUN**	**JUL**	**AUG**	**SEP**	**OCT**	**NOV**	**DEC**	**ACES**
FLUA(H1N1)	FLUB	1·36	1·59	1·95	x	x	x	x	x	x	x	x	x	1·67
FLUA(H3N2)	FLUB	1·35	1·48	1·29	1·53	x	x	x	x	x	x	x	x	1·39
FLUB	HPIV-3	x	1·38	1·34	1·2	x	x	x	x	x	x	x	x	1·34
FLUA-generic	HMPV	1·31	1·23	1·4	x	x	x	x	x	x	x	x	x	1·30
FLUA(H1N1)	RV	1·14	1·16	1·56	1·34	x	x	x	x	x	x	x	x	1·29
FLUA(H3N2)	FLUA(H1N1)	0·58	1·74	1·26	x	x	x	X	x	x	x	x	x	1·29
HPIV-3	RV/EV	x	x	x	x	1·15	x	x	x	x	x	x	x	1·15
FLUA-generic	HRSV	1·29	1·07	0·99	1·18	x	x	x	x	x	x	x	1·42	1·15
FLUA-generic	RV	0·91	1·25	1·2	∞	x	x	x	x	x	x	x	∞	1·14
FLUA-generic	HPIV-3	1·29	1·11	0·95	x	x	x	x	x	x	x	x	1·27	1·14
HPIV-1	HRSV	0·88	x	x	x	x	x	x	x	x	x	x	1·45	1·10
FLUA-generic	HPIV-2	1·42	0·87	x	x	x	x	x	x	x	x	x	x	1·08
FLUA(H3N2)	RV	1·16	0·99	1·03	0·98	x	x	x	x	x	x	x	1·07	1·04
HPIV-generic	HRSV	1·02	x	x	x	x	x	x	x	x	x	x	1·06	1·04
FLUA(H1N1)	HCoV-E229	x	1·02	x	x	x	x	x	x	x	x	x	x	1·02
HPIV-3	EV	x	x	x	x	∞	0·84	1·01	1·22	x	x	x	x	1·01
FLUA-generic	HPIV-1	1	∞	x	x	x	x	x	x	x	x	x	x	1·00
FLUA(H1N1)	HRSV	1·01	0·9	1	0·98	x	x	x	x	x	x	x	x	0·97
FLUA(H1N1)	HPIV-3	x	0·95	x	x	x	x	x	x	x	x	x	x	0·95
HPIV-generic	RV	x	x	x	x	1·02	x	x	x	1·33	0·63	0·87	x	0·94
FLUB	HCoV-E229	x	x	0·92	x	x	x	x	x	x	x	x	x	0·92
FLUB	HMPV	x	0·77	0·99	1·15	x	x	x	x	x	x	x	x	0·91
FLUA(H3N2)	HBoV	0·69	0·9	1·09	x	x	x	x	x	x	x	x	x	0·90
FLUB	HRSV	0·85	0·84	0·99	0·84	x	x	x	x	x	x	x	0·93	0·88
FLUA(H3N2)	HCoV-NL63	x	0·86	x	x	x	x	x	x	x	x	x	x	0·86
FLUA(H3N2)	HMPV	0·75	1·08	0·69	x	x	x	x	x	x	x	x	x	0·86
FLUA(H1N1)	HCoV-OC43	x	0·85	x	x	x	x	x	x	x	x	x	x	0·85
FLUA(H3N2)	HPIV-3	0·9	0·63	1·06	x	x	x	x	x	x	x	x	x	0·85
HMPV	HRSV	0·77	0·81	0·98	0·72	x	x	x	x	x	x	x	0·9	0·84
HPIV-3	HMPV	x	x	0·67	1·2	0·65	x	x	x	x	x	x	x	0·82
FLUA(H1N1)	RV/EV	x	0·82	x	x	x	x	x	x	x	x	x	x	0·82
FLUA(H3N2)	HRSV	0·75	0·87	0·77	1	x	x	x	x	x	x	x	0·71	0·81
FLUA-generic	HCoV-OC43	1·06	0·65	x	x	x	x	x	x	x	x	x	x	0·81
FLUA(H1N1)	HBoV	x	0·8	x	x	x	x	x	x	x	x	x	x	0·80
FLUB	RV	x	0·58	0·87	0·99	∞	x	x	x	x	x	x	x	0·77
FLUA(H1N1)	HMPV	x	0·89	0·59	x	x	x	x	x	x	x	x	x	0·76
FLUA(H3N2)	HAdV	0·75	0·62	0·89	x	x	x	x	x	x	x	x	x	0·75
HPIV-3	HAdV	x	x	x	x	0·87	0·47	x	x	x	x	x	x	0·71
FLUB	HCoV-NL63	x	x	0·69	x	x	x	x	x	x	x	x	x	0·69
HPIV-4	RV	x	x	x	x	x	x	x	x	0·62	0·68	0·67	x	0·66
HRSV	EV	0·54	0·56	0·83	1·05	x	x	x	x	x	x	x	0·37	0·66
FLUA-generic	HBoV	x	0·66	x	x	x	x	x	x	x	x	x	x	0·66
HMPV	RV	0·88	0·37	0·51	0·62	0·6	0·99	1·31	x	x	0·85	0·53	0·64	0·65

FLUA(H1N1)	HAdV	0·75	0·53	0·68	x	x	x	x	x	x	x	x	x	0·64
HRSV	RV	0·62	0·56	0·58	0·79	0·57	x	x	x	x	0·74	0·49	0·78	0·63
HPIV-3	RV	0·72	0·66	0·44	0·52	0·65	0·68	0·58	0·81	0·79	0·75	0·58	0·42	0·61
HPIV-3	HRSV	0·42	0·55	0·65	0·85	0·61	x	x	x	x	x	x	0,64	0·59
FLUA(H1N1)	HCoV-NL63	x	0·95	x	x	x	-1·41	x	x	x	x	x	x	0·58
FLUA-generic	HAdV	0·75	0·44	0·62	x	x	x	x	x	x	x	x	x	0·58
HPIV-1	RV	x	x	x	x	x	x	x	x	0·58	0·58	0·59	x	0·58
FLUA-generic	HCoV-NL63	x	0·58	x	x	x	x	x	x	x	x	x	x	0·58
RV	EV	x	x	x	x	x	x	0·48	0·7	0·53	x	x	x	0·56
RV	HCoV-E229	x	0·67	0·43	0·44	0·59	x	x	x	x	x	x	x	0·54
FLUB	HAdV	x	x	0·49	0·63	x	x	x	x	x	x	x	x	0·54
FLUA-generic	EV	x	0·53	x	x	x	x	x	x	x	x	x	x	0·53
FLUB	RV/EV	x	0·52	x	x	x	x	x	x	x	x	x	x	0·52
HRSV	HAdV	0·45	0·37	0·4	0·83	0·84	x	x	x	x	x	x	0·51	0·50
FLUA-generic	HCoV-E229	x	0,5	x	x	x	x	x	x	x	x	x	x	0·50
HPIV-2	HRSV	x	x	x	x	x	x	x	x	x	x	x	0·49	0·49
HPIV-2	RV	x	x	x	x	x	x	x	x	x	0·47	0·5	x	0·48
RV	HCoV-OC43	0·37	x	0·52	0·58	x	x	x	x	x	0·55	0·36	0·4	0·46
RV	HAdV	x	x	x	x	0·36	0·31	0·48	0·72	x	x	0·37	x	0·43
FLUA(H3N2)	HCoV-OC43	0·42	x	x	x	x	x	x	x	x	x	x	x	0·42
HRSV	HCoV-E229	0·57	0·3	x	x	x	x	x	x	x	x	x	x	0·42
RV	HBoV	x	X	x	x	x	x	x	x	x	x	0·39	0·35	0·37
HRSV	HCoV-OC43	0·3	0·36	0·4	x	x	x	x	x	x	x	X	0·19	0·33
HRSV	HCoV-NL63	x	0·29	0·33	x	x	x	x	x	x	x	x	x	0·31
HAdV	HBoV	x	-0·29	x	x	x	x	x	x	x	x	x	x	-0·29
HCoV-NL63	HCoV-HKU1	x	x	-0·82	x	x	x	x	x	x	x	x	x	-0·82
HCoV-OC43	HCoV-E229	x	x	x	x	x	x	x	x	x	-1·03	x	x	-1·03
HPIV-1	HCoV-NL63	x	x	x	x	x	x	-1·12	x	x	x	x	x	-1·12
FLUA(H3N2)	HPIV-4	x	x	x	x	-1·39	x	x	x	x	x	x	x	-1·39

Appendix Table 2. Coinfection interactions. The table shows the 73 coinfections which exhibited at least one month with significant coinfection exclusion score (CES). A positive average coinfection exclusion score (ACES) indicates an exclusion interaction while a positive ACES an affinity interaction. The score assumes a value of 1 if coinfections are ten times less likely than expected by chance and a value of -1 if they are ten times more likely. The X indicates months where the CES were no significant. The symbol ∞ indicates zero coinfections in that month regarding the virus pair.
